# Epstein-Barr Virus-Encoded Small RNAs (EBERs) Are Present in Fractions Related to Exosomes Released by EBV-Transformed Cells

**DOI:** 10.1371/journal.pone.0099163

**Published:** 2014-06-04

**Authors:** Waqar Ahmed, Pretty S. Philip, Saeed Tariq, Gulfaraz Khan

**Affiliations:** 1 Department of Microbiology and Immunology, College of Medicine and Health Sciences, United Arab Emirates University, Al Alin, United Arab Emirates; 2 Department of Anatomy, College of Medicine and Health Sciences, United Arab Emirates University, Al Alin, United Arab Emirates; Gustave Roussy, France

## Abstract

Epstein-Barr virus (EBV) is an oncogenic herpesvirus associated with a number of human malignancies of epithelial and lymphoid origin. However, the mechanism of oncogenesis is unclear. A number of viral products, including EBV latent proteins and non-protein coding RNAs have been implicated. Recently it was reported that EBV-encoded small RNAs (EBERs) are released from EBV infected cells and they can induce biological changes in cells via signaling from toll-like receptor 3. Here, we investigated if these abundantly expressed non-protein coding EBV RNAs (EBER-1 and EBER-2) are excreted from infected cells in exosomal fractions. Using differential ultracentrifugation we isolated exosomes from three EBV positive cell lines (B95-8, EBV-LCL, BL30-B95-8), one EBER-1 transfected cell line (293T-pHEBo-E1) and two EBV-negative cell lines (BL30, 293T-pHEBo). The identity of purified exosomes was determined by electron microscopy and western blotting for CD63. The presence of EBERs in cells, culture supernatants and purified exosomal fractions was determined using RT-PCR and confirmed by sequencing. Purified exosomal fractions were also tested for the presence of the EBER-1-binding protein La, using western blotting. Both EBER-1 and EBER-2 were found to be present not only in the culture supernatants, but also in the purified exosome fractions of all EBV-infected cell lines. EBER-1 could also be detected in exosomal fractions from EBER-1 transfected 293T cells whilst the fractions from vector only transfectants were clearly negative. Furthermore, purified exosomal fractions also contained the EBER-binding protein (La), supporting the notion that EBERs are most probably released from EBV infected cells in the form of EBER-La complex in exosomes.

## Introduction

Epstein-Barr virus (EBV) is arguably one of the best studied oncogenic viruses associated with human malignancies. EBV readily infects human B-lymphocytes, both *in vivo* and *in vitro* [1], [2]. EBV infection of B-cells *in vitro* leads to their immortalization and establishment of lymphoblastoid cell line (LCL). In these cells, the virus establishes a latent infection in which 6 nuclear antigens (EBNA-1, EBNA-2, EBNA-3a, EBNA-3b, EBNA-3c, EBNA-LP), three virus-encoded latent membrane proteins (LMP-1, LMP-2a, LMP-2b) and two non-protein encoding RNAs (EBER-1 and EBER-2) are expressed without killing the cell [3]. It is widely held that some of these EBV products play a central role in EBV-mediated oncogenesis [4]–[6].

EBER-1 and EBER-2 are non-polyadenylated and non-protein coding RNA molecules [7]. These polymerase III transcripts (166 & 172 bp respectively) are highly expressed (>10^6^ copies per cell) [7], [8] in all EBV latently infected cells and are often used as targets for the detection of EBV in histological material using *in situ* hybridization [9], [10]. At the sequence level, EBER-1 and EBER-2 are only 54% homologous, although both are highly conserved amongst EBV strains. In spite of their small size, both EBERs exhibit a well-defined secondary structure comprising of intermolecular base-pairing and several stem-loops [8], [11]. Moreover, both form RNA-protein complexes by binding to cellular proteins, at least 5 of which have been identified, namely: the lupus antigen La protein [12], [8], the ribosomal protein L22 [13], [14], the double-stranded RNA-dependent protein-kinase R (PKR) [15], the retinoic acid inducible gene 1 (RIG-1) [16] and more recently the AU-rich element binding factor-1 (AUF-1) [17]. In spite of their abundance and well characterized structure, the physiological function and mechanism of action of EBERs is poorly understood. Although EBERs are not essential for EBV-immortalization of B-cells *in vitro* [18], a growing body of evidence suggests that they play a role in one or more of the following processes: inhibition of apoptosis [19]–[21], increase cell proliferation [22]–[24] and induction of tumor formation [24]–[26]. More recently, it has also been shown that EBER-1, which is the most abundant and most stable of the two [27], is excreted from cells as an RNA-protein complex and is able to induce pro-inflammatory cytokines such as IL-12 via Toll-like receptor 3 (TLR3) [28]. However, the mechanism of EBER excretion remains unknown.

A number of studies have shown that cells infected with EBV actively release exosomes [29]–[32]. Exosomes are diverse bioactive extracellular small membrane vesicles (30–120 nm in size) derived from the cell's endosomal membrane system [33]. Exosomes are generated through the membrane invaginations of multicellular bodies (MVB), which are known to take up material from the cell cytoplasm by inward budding of the MVB membranes [34]. This results in the formation of the intraluminal vesicles (ILVs) that on release are referred as exosomes. Exosomes have been shown to contain a variety of cellular content, including microRNA (miRNA), DNA, proteins and even virus particles [30], [29].

We hypothesized that EBERs are excreted via exosomes [35] which are probably taken up by neighbouring cells by endocytosis [32]. This hypothesis is indeed plausible, considering that the EBER-binding protein La has also been reported to be excreted via exosomes [36]. Moreover, exosomes are now believed to be an important mechanism of transport of numerous small RNA and protein molecules and a means of intercellular communication [37], [38]. In this study we show that both EBER-1 and EBER-2 are present in culture supernatants of EBV-infected cells and are excreted out of the cells in a form that is protected by RNase. Our data supports the notion that EBERs are bound to La-protein and are released in exosomes. Whether EBER carrying exosomes can be taken up by uninfected cells, as has been shown for LMP-1[32], needs further investigation.

## Methods and Materials

### Cell lines and culture

The following established cell lines were used: B95-8 (marmoset EBV-immortalized B-cell line) [39], BL30 and BL30-B95-8 (EBV-negative and positive Burkitt's lymphoma B-cell lines, respectively) (gifts from Prof Martin Rowe, Birmingham University, UK) [40] and 293T (EBV-negative human embryonic kidney cells) (gift from Prof Tahir Rizvi, UAE University) [41]. 293T cells stably transfected with either EBER-1 expression plasmid (pHEBo-E1) or empty plasmid (pHEBo) were also created. In addition to these established cell lines, an EBV positive lymphoblastoid cell line (EBV-LCL) was created by infecting fresh human peripheral blood lymphocytes with EBV as previously described [42]. The study was approved by the Al Ain Medical District Human Research Ethics Committee, AAMD HREC 14/13). B95-8, EBV-LCL, BL30-B95-8 and BL30 were cultured in RPMI-1640 (GIBCO, USA), supplemented with 10% FBS (GIBCO, USA), 100 U/ml penicillin/streptomycin (GIBCO, USA), 50 µg/ml gentamycin (Hyclone, USA) and 1× glutamine (GIBCO, USA). For BL30 and BL30-B95-8, 1 mM Sodium pyruvate, 50 mM α-thioglycerol (Sigma, M-6145) and 10 mM bathocupronic disulfonic acid were also added to the media. 293T cells stably transfected with EBER-1plasmid (pHEBo-E1) or empty plasmid (pHEBo) were cultured in DMEM supplemented with 10% FBS (GIBCO, USA), 100 U/ml penicillin/streptomycin (GIBCO, USA), 50 µg/ml gentamycin (Hyclone, USA) and 1× glutamine (GIBCO, USA) and 150 µg/ml of hygromycin (Hyclone, USA). All cell lines were grown in exosome depleted FBS at 37°C in 5% CO_2_. Exosomes were depleted from FBS by ultracentrifugation as previously described [43].

### Transfection of 293T cells with EBER-1

An expression plasmid containing EBER-1 (kind gift of Prof Paul Farrell, Imperial College London, UK) was created by cloning the entire sequence of EBER-1 into BglII/HindIII restriction sites directly adjacent to the H1 promoter in pHEBo-H1 plasmid [44].The pHEBo-H1 plasmid also contains oriP, hygromycin B and ampicillin resistance genes for selection. For stable transfection of 293T cells with EBER-1 plasmid (293T-pHEBo-E1) or empty plasmid (293T-pHEBo), we used the calcium phosphate method. Briefly, 0.4×10^6^ cells/well were transfected with 3 µg of plasmid DNA. After 48 hours post transfection, cells were selected for hygromycin resistant colonies by trypsinizing the cells and plating them in media containing 150 µg/ml of hygromycin B. Once the resistant colonies emerged (after about 10 days), individual colonies were picked up and cultured until they reached confluency. These stable cell lines were subsequently used for some of the downstream experiments.

### Isolation of exosomes

Exosomes were isolated from EBV positive (B95-8, EBV-LCL, BL30-B95-8), EBV negative (BL30, 293T-pHEBo) and EBER-1 transfected cells (293T-pHEBo-E1) by differential ultra-centrifugation as previously described [43]. Briefly, 2×10^7^cells in late log phase were used for exosomes extraction. Cell viability was checked by trypan blue exclusion and cultures with viabilities above 95% were used. For each cell line, culture supernatant was centrifuged at 2000xg at 4°C for 20 minutes. The supernatant was carefully removed (leaving behind approximately 1.5 ml of cell pellet/media), and centrifuged at 10,000xg for 30 min at 4°C using SW32 Ti rotor (Beckman, Fullerton, USA). The supernatant was collected and centrifuged at 100,000xg for 70 minutes at 4°C. The supernatant was carefully aspirated off and the exosome containing pellets were washed by resuspending them in PBS and centrifuging at 100,000xg for 70 minutes at 4°C. The final exosome pellets were resuspended in 50 µl of PBS and either stored in −80°C or used immediately for down-stream experiments.

### Transmission electron microscopy on exosomes

10 µl of exosome suspension in 1xPBS was dried onto freshly glow discharged 200 mesh formvar-carbon-coated copper grids (Ted Pella, Redding, CA), negatively stained with 2% aqueous uranyl acetate and observed with a Philips CM10 transmission electron microscope (TEM) (Philips, Eindhoven, The Netherlands). Images were captured with a side mounted 1K AMT Advantage digital camera (Advanced Microscopy Techniques, Corp. Woburn, MA).

### Western blotting for CD63 and La protein in exosomes

To confirm the identity of exosomes visualized in TEM, exosomal proteins were extracted and western blotting performed for CD63 (marker of exosomes) [31] using anti-CD63 monoclonal antibody (ab8219, Abcam, UK) under non-reducing conditions as recommended by the manufacturer. To determine if the EBER-binding protein La was excreted in exosomes, western blotting was performed using anti-La monoclonal antibody (sc-166274, Santacruz, USA) under reducing conditions. The total protein concentration of the exosomal fraction was determined by the Bradford protein assay using Bio-Rad Protein Assay Dye Reagent Concentrate (Bio-Rad Laboratories, Hercules, USA). Between 25–35 µg of exosomal proteins and up to 200 µg of cellular proteins were used in each assay.

### RNA extraction for RT-PCR

Total RNA was extracted from cells, culture supernatants and purified exosomes using TRIzol reagent (Invitrogen, USA) and quantified using the Nanodrop instrument. For extraction of RNA from cells and culture supernatants, cells were grown to a density of 0.5–1.0×10^6^cells/ml and RNA extracted from a total of 0.5×10^6^ cells or from 1 ml of cell free culture supernatants. For extraction of RNA from exosomes, purified exosomes from culture supernatants corresponding to approximately 2×10^7^cells was used. 1 µg of RNA was reverse transcribed to cDNA using the Reverse Transcription Kit (Promega, USA) following the manufacturer's instructions. Before reverse transcription, all RNA samples were routinely treated with DNase I (Promega, USA) to remove any contaminating genomic/viral DNA and tested for EBER amplification using PCR. Similarly, purified exosomes were treated with RNase A prior to RNA extraction, as previously described [45], to ensure that extra-exosomal RNA was removed.

### RT-PCR for EBERs and sequencing

Reverse transcriptase-PCR (RT-PCR) was performed for EBER-1 and EBER-2 using the following set of primers:

EBER-1 forward primer: 5′- ccc aga tct AGG ACC TAC GCT GCC C - 3′

EBER-1 reverse primer: 5′- ccc aag ctt AAA ACA TGC GGA CCA CCA GC - 3′

EBER-2 forward primer: 5′- ccc aga tct AGG ACA GCC GTT GCC CTA GT- 3′

EBER-2 forward primer: 5′- ccc aag ctt AAA AAT AGC GGA CAA GCC GAA T- 3′

Note that each primer is flanked by a 9 nucleotide sequence (indicated in lower case) for restriction enzymes and hence the expected RT-PCR amplification product is 184 bp for EBER-1 and 190 bp for EBER-2. All RT-PCR reactions were carried out using 1U of *Taq* polymerase (Applied Biosystems), 0.5 mM dNTPs, 1× PCR reaction buffer, 2 mM MgCl_2_ and 10 pmol of each forward and reverse primer and 1–2 µl of cDNA in 30 µl reactions. PCR was performed by an initial 5 minutes denaturation at 94°C followed by 30 cycles of 94°C for 1 min s, 51°C/46°C (EBER-1/EBER-2 respectively) for 30 seconds and 72°C for 60 seconds with a final elongation at 72°C for 7 minutes. Each PCR run included at least one positive (EBER-1 or EBER-2 plasmid DNA) and negative control (sterile water instead of template). PCR reactions were carried out using an Applied Biosystems thermal cycler PCR System 2700. Amplified products were visualized on 2% agarose gel stained with ethidium bromide.

PCR amplified products were sequenced using the ABI Genetic Analyzer (3130×1), following the protocol of ABI Big Dye Terminator Reaction (Applied Biosystems Inc., CA, USA). The sequence data was analyzed using sequence analysis software v5.3 (ABI, CA, USA) and compared with the B95-8 EBV reference sequences in the GenBank, accession number V01555.2.

## Results

### Both EBER-1 and EBER-2 are released into the culture supernatant of EBV-immortalized cells

**Figure 1:**
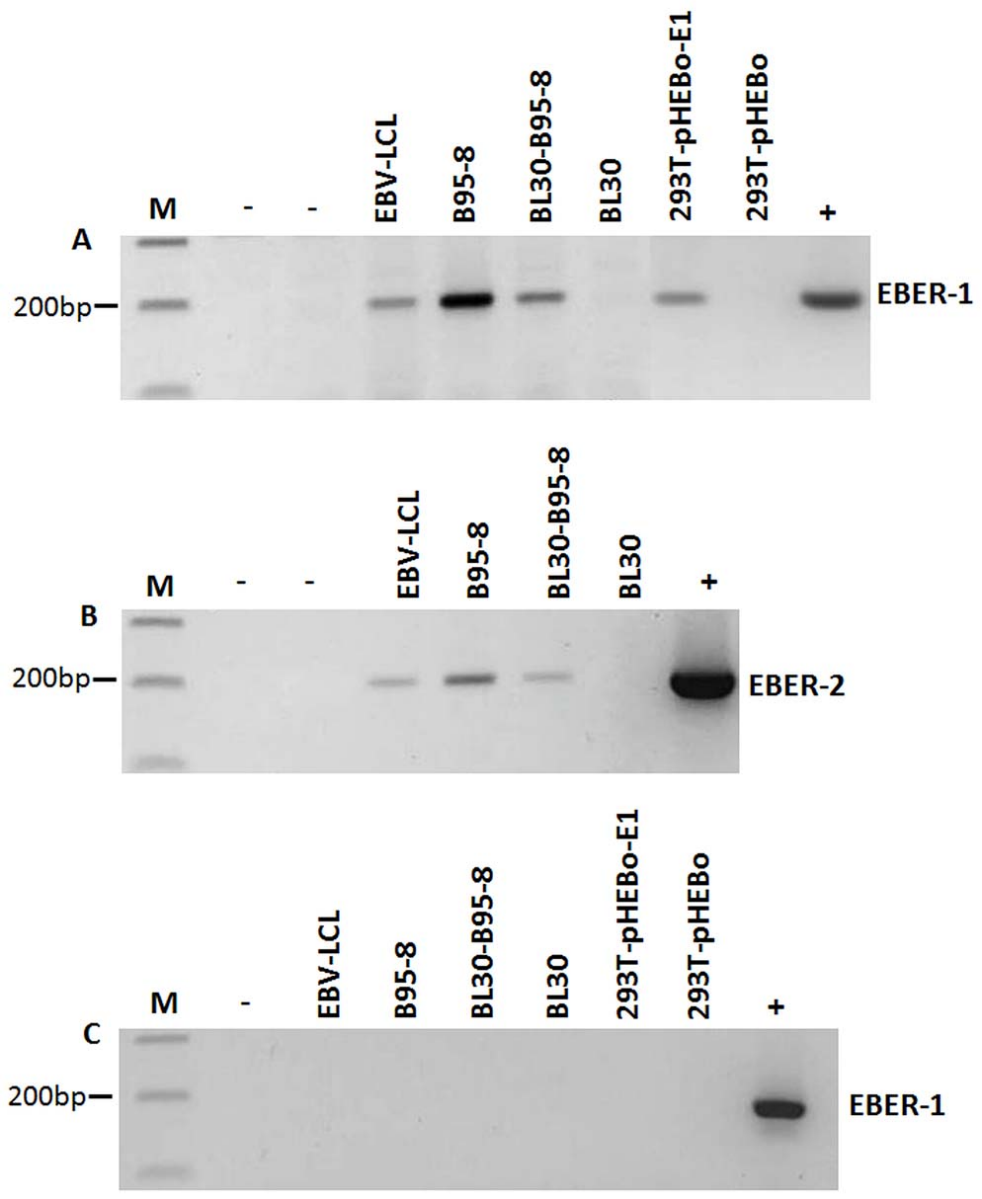
Reverse transcriptase PCR for EBERs on genomic RNA. RT- PCR was carried out on genomic RNA extracted from three EBV positive cell lines (B95-8, EBV-LCL and BL30-B95-8), 293T cells stably transfected with EBER-1 plasmid (293T-pHEBo-E1), and two EBV negative cell lines (BL30 and 293T stably cells transfected with empty plasmid (293T-pHEBo)). cDNA from these cells was subjected to 30 rounds of PCR amplification for (A) EBER-1 and (B) EBER-2 and the amplified products were visualized on a 2% agarose gel. Positive (+) (EBER-1 or EBER-2 plasmid DNA) and negative (−) (sterile water) controls are indicated. All three EBV positive cell lines showed specific amplification of EBER-1 and EBER-2. BL30 and 293T-pHEBo cells were clearly negative. Furthermore, EBER-1 specific amplification was also seen in EBER-1 transfected 293T-pHEBo-E1 cells. (C) To ensure that the EBER-amplification seen in Figure 1A and 1B was not due to EBV DNA contamination, PCR was performed for EBER-1 and EBER-2 on DNase treated RNA samples prior to reverse transcription. No amplification was seen, clearly indicating the absence of any contaminating DNA (results shown for EBER-1 only).

**Figure 2:**
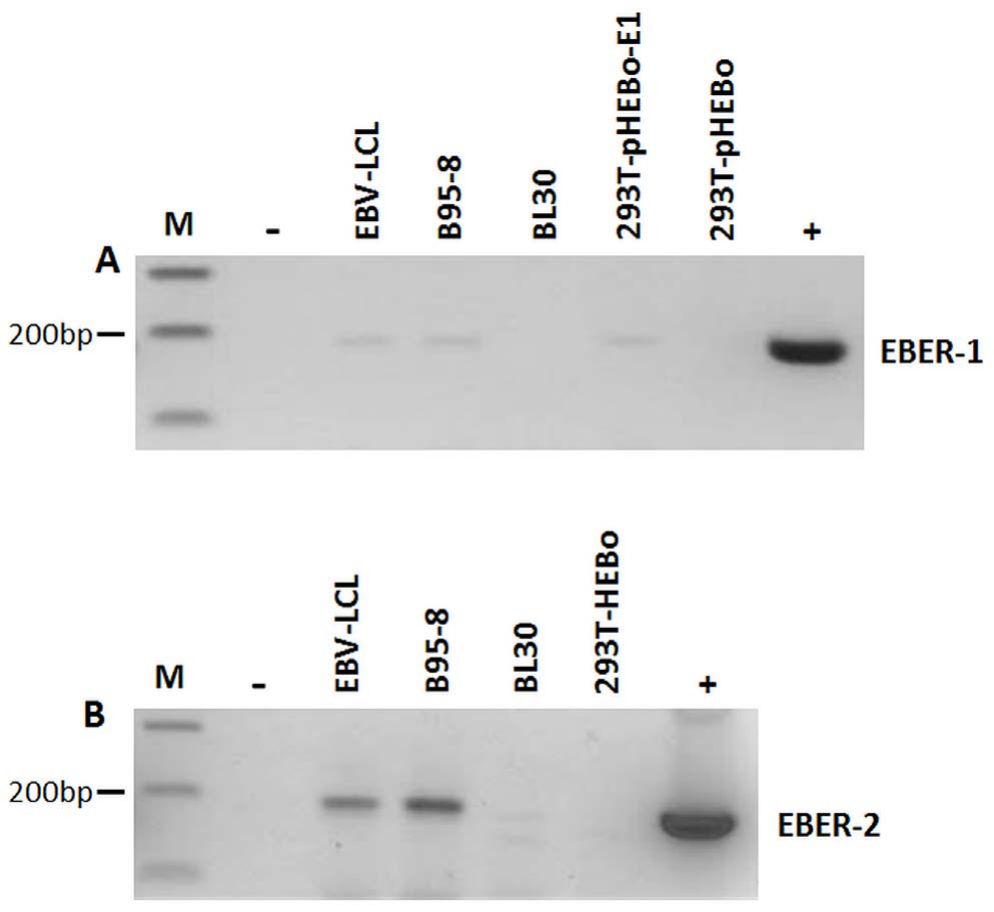
Reverse transcriptase PCR for EBERs on culture supernatants. RNA was extracted from 1-PCR was carried out on RNA extracted from culture supernatant of EBV positive cell lines (EBV-LCL, B95-8), 293T cells stably transfected with EBER-1 (293T-pHEBo-E1) and two EBV negative cell lines (BL30 and 293T cells stably transfected with empty plasmid (293T-pHEBo)). cDNA was subjected to 30 rounds of PCR amplification for (A) EBER-1 and (B) EBER-2. Positive (+) (EBER-1 or EBER-2 plasmid DNA) and negative (−) (sterile water) controls are indicated. Both EBER-1 and EBER-2 were amplified from EBV positive cell lines (B95-8 and EBV-LCL) whilst the EBV negative cell lines BL30 and 293T-pHEBo were negative. EBER-1 transfected 293T-pHEBo-E1 also showed specific amplification.

RT-PCR on RNA extracted from EBV-infected cell lines (B95-8, EBV-LCL and BL30-B95-8) was consistently positive for both EBER-1 and EBER-2, but not in non-infected cells (BL30, 293T-pHEBo) (Figure 1A and 1B). All our RNA extractions were DNase treated and tested for EBERs using PCR prior to reverse transcription. No EBER amplification was seen in any of these samples (Figure 1C). Thus, the EBER-positivity presented in Figures 1A and 1B cannot be due to EBV DNA or plasmid contamination. Furthermore, stable transfection of 293T cells with EBER-1 expression plasmid (293T-pHEBo-E1) also resulted in EBER-1 specific amplification (Figure 1A). We next isolated RNA from 1 ml of culture supernatants of late log phase growing cells and performed RT-PCR for EBER-1 and EBER-2. The results indicated that both EBERs were excreted, albeit at low levels, from EBV-immortalized cells into the culture supernatant (Figure 2A and 2B). These findings confirm the previous report by Iwakiri et al. [28] showing that EBER-1 is present in the culture supernatants of EBV infected cells. However, in contrast to Iwakiri et al.'s study, we also detected the presence of EBER-2 in the culture supernatants of EBV infected cells. The experiment was repeated several times and the same result was obtained.

### Purification of exosomes from EBV-infected and non-infected cells

**Figure 3:**
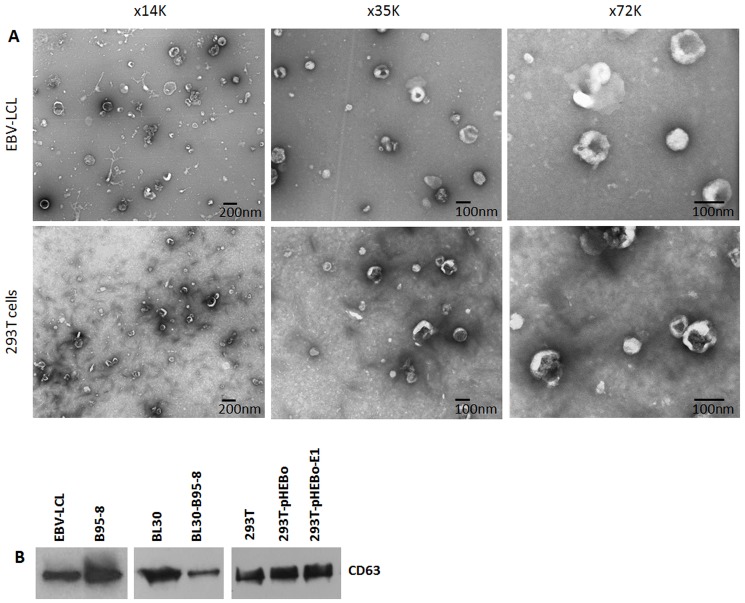
Transmission electron microscopy and western blot for CD63 on exosomal fractions. Exosomes were isolated using differential ultracentrifugation and examined using transmission electron microscopy. (A) Nanovesicles with typical size (50–120 nm) and morphology resembling exosomes were observed in isolates from both EBV positive (EBV-LCL) and negative (293T) cells. (B) Western blotting for the exosomal marker CD63, confirmed the identity of these nanovesicles to be exosomes.

We have recently proposed that EBERs are released from EBV-infected cells by an active process involving exosomes [35]. To investigate this, we isolated exosomes from culture supernatants of EBV-infected and non-infected cells using differential centrifugation [43]. Purified exosome suspensions were examined by transmission electron microscopy (TEM) at various magnifications. Vesicles with characteristics of exosomes were clearly visible in isolates from both EBV-infected and non-infected cells (Figure 3A). Morphologically, exosomes from both EBV-infected and non-infected cells were indistinguishable and varied in size from 50–120 µm. Since we used exosome depleted FBS in our culture media, the exosomes isolated must be from cells and not from FBS which is also known to contain exosomes [46]. To further confirm the identity of our isolated exosomes, we performed western blotting for CD63, a marker of exosomes [31]. The results confirmed that the isolates were indeed exosomes (Figure 3B) (also see Figure S2)

### Detection of EBERs and EBER binding protein La in exosomal fractions of EBV-immortalized cells

**Figure 4:**
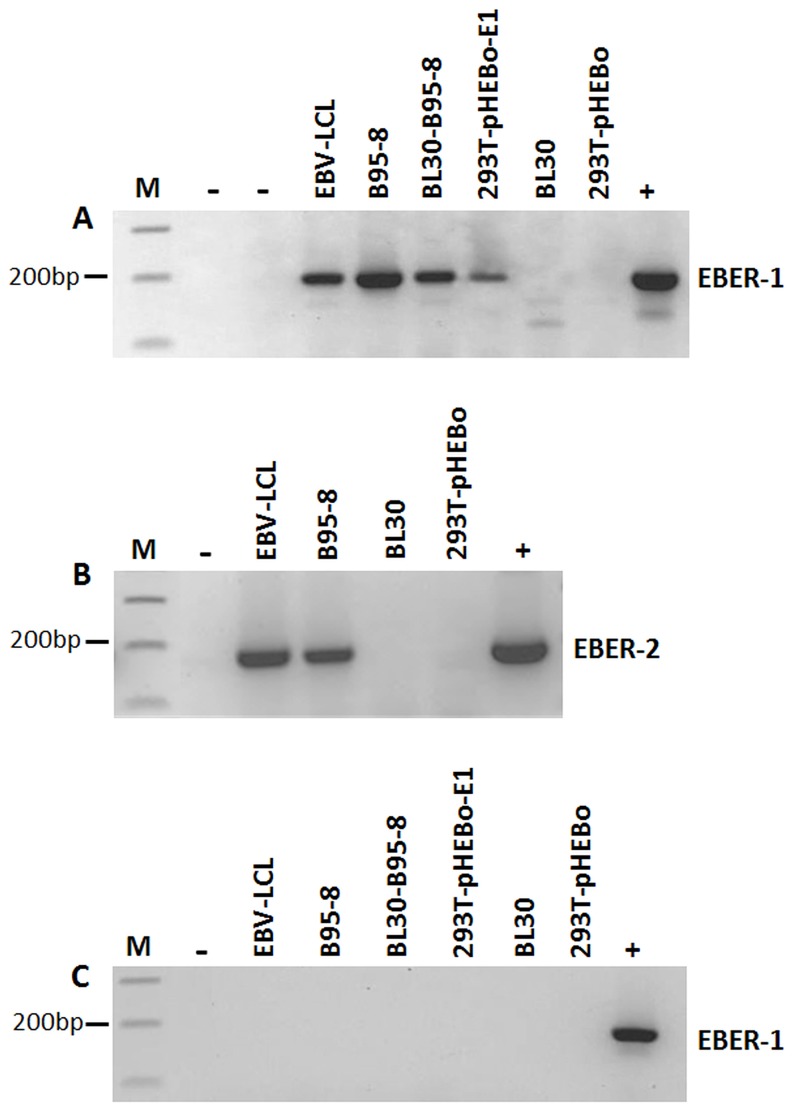
Reverse transcriptase PCR for EBERs on exosomal RNA. RT-PCR for (A) EBER-1 and (B) EBER-2 on the purified exosomes from EBV-positive and negative cells, gave positive amplification only in EBV infected cell lines. EBER-1 stably transfected 293T cells (293T-pHEBo-E1) were also positive for EBER-1, but the amplification signal was weaker than that seen with EBV-infected cell lines. (C) DNase treated RNA samples prior to reverse transcription consistently gave negative results, indicating that the amplification signals seen in Figure A and B were not due to DNA contamination (results shown for EBER-1 only). Positive (+) (EBER-1 or EBER-2 plasmid DNA) and negative (−) (sterile water) controls are indicated.

RNA isolated from the purified exosomes was treated with DNase, reverse transcribed to cDNA and RT-PCR performed for EBER-1 and EBER-2. Bands corresponding to the expected size of the amplification products were clearly visible in the agarose gel for both EBER-1 and EBER-2 (Figure 4A and 4B). EBER positivity was only seen for exosomes isolated from EBV-infected cell lines and not from the EBV-negative cells lines (Figure 4A and 4B). Similarly, no EBER amplification was seen when exosomal RNA was used prior to reverse transcription, indicating that the EBER positive signals were not due to any contaminating DNA (4C). To verify that the PCR amplified products visible in the agarose gels were indeed EBER-1 and EBER-2, the products were purified from the agarose gels and subsequently sequenced using the ABI Genetic Analyzer (3130×1) and the protocol of ABI Big Dye Terminator Reaction (Applied Biosystems Inc., CA, USA) as previously described [47]. The sequence data confirmed the PCR amplified products to be EBER-1 and EBER-2 (accession number V01555.2).

Exosomes isolated from 293T cells stably transfected with EBER-1 (293T-pHEBo-E1 cells) also gave a positive signal (Figure 4A). However, the EBER-1 amplification signal from 293T-pHEBo-E1 cells was weaker than that seen for EBV-infected cells (Figure 4A). The weak signal from 293T-pHEBo-E1 cells may be due to a lower level of expression of EBER-1 in these cells, as compared to EBV infected cells (unpublished data). This in turn could be due to the fact that transfected cells do not have any of the other EBV latent proteins, in particular EBNA1, to enhance EBER expression [48]. Exosomes isolated from 293T cells stably transfected with empty plasmid (293T-pHEBo cells) were consistently negative for both EBERs (Figure 4A and 4B).

**Figure 5:**
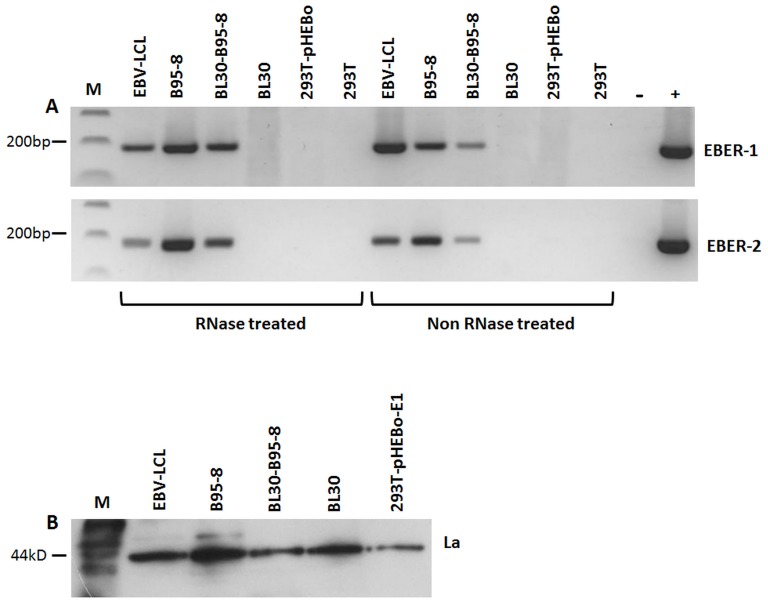
Detection of EBERs and EBER binding protein La in exosomal fractions. (A) RNase A treatment of purified exosomes prior to RNA extraction and RT-PCR did not abolish EBER amplification signal, suggesting that EBERs are present in exosomes and not in the extra-exosomal fraction. (B) To determine if the EBER-1 binding protein La was present in exosomes, 25 µg of exosomal protein fraction was separated by 10% SDS PAGE and immunoblotted using anti-La monoclonal antibodies. Exosomal fractions from all cell lines clearly showed presence of La protein, irrespective of whether they were EBV infected or not.

To ascertain that the EBER positivity was from exosomal fraction and not extra-exosomal EBER contaminants, we treated purified exosomes with RNase A prior to RNA extraction. This step will remove any non-exosomal RNAs [45]. RT-PCR performed on RNase treated samples remained EBER positive, indicating that the positive signals were from exosomal fraction (Figure 5A). To shed light on the possible mechanism of EBER excretion, we examined the presence of La protein in the exosome fractions. La is one of the cellular proteins known to bind to EBER-1 [12]. Moreover it has been shown to be excreted from cells via exosomes [36]. Western blotting performed on purified exosomal proteins from EBV-positive and negative cells, clearly showed that this protein was present in all exosomal fractions (Figure 5B and Figure S1).

## Discussion

Epstein-Barr virus-encoded small RNAs (EBERs) are by far the most abundant viral transcripts expressed in infected cells [7], [8]. Moreover, they are expressed in all three patterns of EBV latency. The function of these non-protein coding polymerase III transcripts remains elusive. A number of reports indicate that they play a role in cell proliferation and inhibition of apoptosis, and hence contribute to EBV-mediated oncogenesis [24], [25], [49]. Which of the two EBERs play a more central role in these processes remains controversial [26], [50]. More recently, it was shown that EBER-1 and not EBER-2 is abundantly excreted from EBV-infected cells [28]. Furthermore, the extracellular EBER-La complex was shown to induce type 1 IFN and pro-inflammatory cytokines via toll-like receptor 3 (TLR3) signaling [28]. It has been proposed that EBER-1 is released from infected cells by the active secretion of La via exosomes [28], [35]. Exosome-mediated release would not only provide stability to EBERs from degradation by nucleases, but also a means of transport to uninfected cells. Indeed, exosomes are now considered to be an essential cellular process for the exchange and traffic of cellular cargo such as proteins and miRNAs [51], [52]. In the context of EBV, it has been shown that the EBV-oncoprotein LMP-1 and a number of miRNAs are released via exosomes and they can induce biological changes in neighboring cells [30], [53], [54]. Here, we add EBERs to this growing list of cellular and viral products that are excreted in exosomes. We report the presence of both EBER-1 and EBER-2, not only in the culture supernatant of EBV infected cells, but importantly in purified exosomal fraction. Our results also indicated the presence of EBER-1 in the exosomal fraction of 293T cells stably transfected with EBER-1, though the level was not as high as that seen in EBV-infected cells. This might be due to the lower expression of EBER-1 in transfected cells as compared to the viral infected cells (unpublished data). The lower level of EBER-1 expression in EBER-1 transfected 293T may be due to the absence of EBNA1 to enhance EBER expression [48]. The fact that RNase treatment of purified exosomes prior to RNA extraction did not abolish EBER positivity indicates that EBERs are present within the exosomes and protected from RNase digestion. We also show the presence of the EBER binding protein La in the exosomal fractions, confirming a previous report [36]. La protein is one of the most abundant proteins in the human cell and it has been shown to bind to all newly synthesized RNAs in the cell [55]. Functional analysis suggests that La acts as a molecular chaperon for small RNA molecules, providing them stability and protection from exonuclease digestion [8]. Our results showing the presence of both EBERs and La in the same exosomal fraction, highly suggests that EBERs are piggybacking the La protein excretion pathway. Previous reports showing the presence of both EBERs and La at high levels in the cell nucleus suggests that this interaction may take place in the nucleus. However, EBERs are not exclusive to the nucleus and studies reporting their presence in the cytoplasm has also been reported [56]. Indeed, EBERs were first identified from cytoplasmic preparations of EBV infected Burkitt's lymphoma cells, indicating that they were present in the cytoplasm [7]. Furthermore, recent data indicates that EBERs may also be excreted out of EBV infected cells, existing in the extracellular environment as EBER-La complex [28]. Our data suggest that EBERs may be excreted as EBER-La complex out of infected cells utilizing the exosome pathway. How important La binding is for EBER excretion needs further investigation. Deletions of La binding site in EBERs may provide some answers.

Our findings support the previous report showing high levels of EBER-1in EBV-infected culture supernatants [28]. However, in contrast to this report, we also found clearly detectable levels of EBER-2 in the culture supernatants. The differences in the two results may be due to differences in the cell lines used. The shorter half-life of EBER-2 as compared to EBER-1 could also be an additional variable. Furthermore, we used 30 cycle PCR for amplification of EBER-2 as compared to 25 used by Iwakiri et al. [28].

Future studies need to address the biological effects of exosomal form of EBERs on non-infected surrounding cells. A few recent studies indicate that culture supernatant from EBV infected cells is biologically active [28], [32]. However, it is not entirely clear whether these biological effects are entirely due to EBERs or other excreted cellular and/or viral components. For example, it is now well established that EBV infected cells release miRNAs via exosomes [45], [57]. These miRNAs play an instrumental role in inducing cell proliferation, inhibition of apoptosis and regulation of viral infection [58]–[60]. Studies have also indicated the presence of LMP-1 in EBV exosomes [53], [61]. LMP-1 is known to have tumorigenic properties and it has the ability to promote cell growth, resistance to apoptosis and induce phenotypic changes in EBV infected cells [62], [63]. In addition to miRNA and LMP-1, a recent study has shown that EBV exosomes also contain mRNA for LMP-1, LMP-2, EBNA-1 and EBNA-2 [57]. It is expected that future studies will shed further light on exosomal contents of EBV infected cells and their impact on surrounding cells.

## Supporting Information

Figure S1 **Western blots for La protein.** Some representative western blot experiments for La protein on (A) cellular extracts and (B) exosomal extracts. Western blot for the detection of La was first optimized on cellular extracts, using up to 200 µg of cellular proteins extracts. However, this was subsequently reduced in later experiments to 100 µg. Anti-La antibody (Santacruz, USA) was used at a dilution of 1∶400 in reducing conditions. For exosomal extracts, we used 25–35 µg of proteins. The hand written annotations, MWM, LCL, BL30+ and BL30- refer to molecular weight marker, EBV-LCL, BL30-B958 and BL30 cell lines respectively – see main manuscript for further details.
(DOCX)

Figure S2 **Western blots for CD63.** Some representative western blots for CD63 on (A) cellular and (B) exosomal extracts. For the detection of CD63 in cellular extracts, 100 µg of proteins was used and for exosomal extracts 25–35 µg of protein was used. Anti-CD63 antibody (Abcam, UK) was used at a dilution 1∶1000 in non-reducing conditions. The hand written annotations refer to the various cell lines used (see main manuscript for further details). Cell lines SL1, SL3 and SL4 (Fig.B right side of the blot) are not part of this study and should not be considered here. Also note that in Fig.B (left side of the blot), exosomal extracts from both freshly prepared and older preparations were used. The relatively weak bands seen for ‘old’ samples appear to be due to protein degradation. Indeed, when freshly prepared exosomal extracts were used, stronger signals were observed e.g. compared 293T old cell extracts (left side of blot) and freshly prepared 293T cell extracts (right side of the blot) (also see Figure 3B in manuscript).
(DOCX)
