# Supplementary figures and images for: Epstein-Barr Virus-Encoded Small RNAs (EBERs) Are Present in Fractions Related to Exosomes Released by EBV-Transformed Cells
Source: PLoS One. 2014 Jun 4;9(6):e99163. doi: 10.1371/journal.pone.0099163 (PMC4045842; doi:10.1371/journal.pone.0099163)

**Figure S1**

**(A) (B)**

**
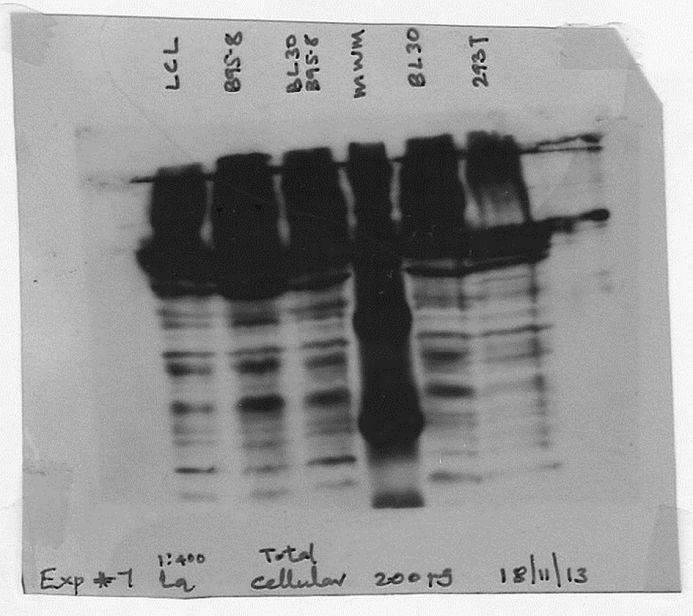

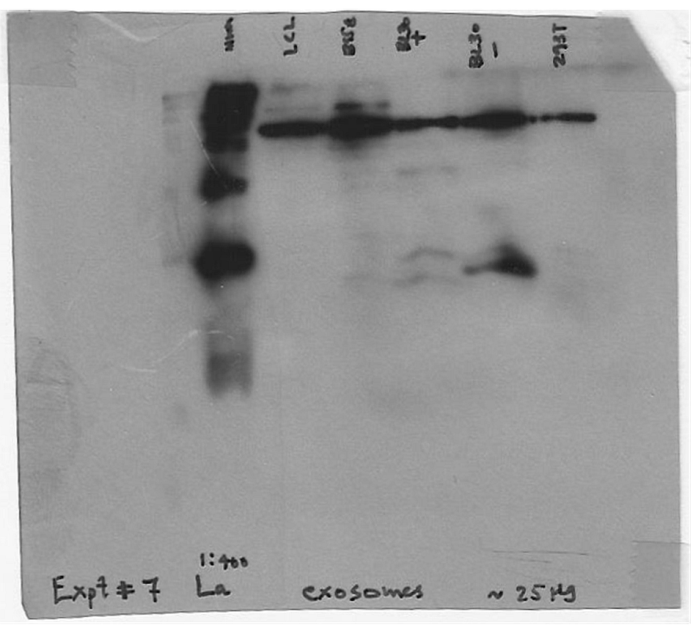
**

Supplement: Figure S1 — Western blots for La protein. Some representative western blot experiments for La protein on (A) cellular extracts and (B) exosomal extracts. Western blot for the detection of La was first optimized on cellular extracts, using up to 200 µg of cellular proteins extracts. However, this was subsequently reduced in later experiments to 100 µg. Anti-La antibody (Santacruz, USA) was used at a dilution of 1∶400 in reducing conditions. For exosomal extracts, we used 25–35 µg of proteins. The hand written annotations, MWM, LCL, BL30+ and BL30- refer to molecular weight marker, EBV-LCL, BL30-B958 and BL30 cell lines respectively – see main manuscript for further details. (DOCX) [file pone.0099163.s001.docx]

**Figure S2**

**(A)**

**
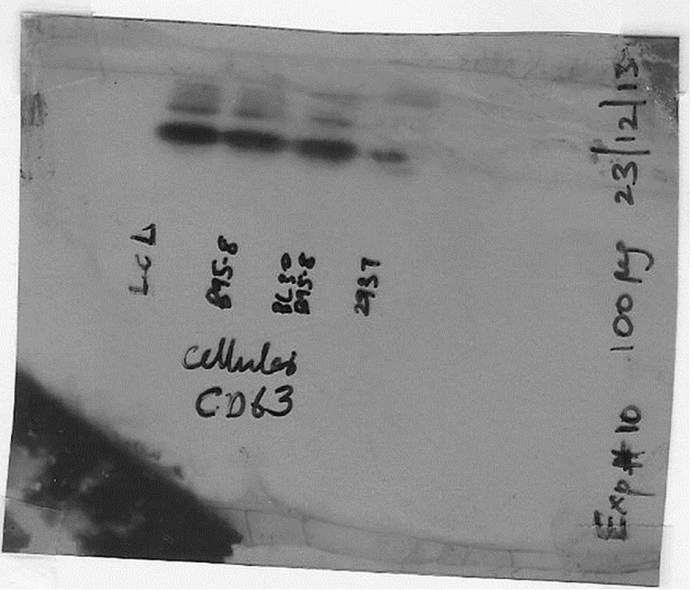
**

**(B)**

**
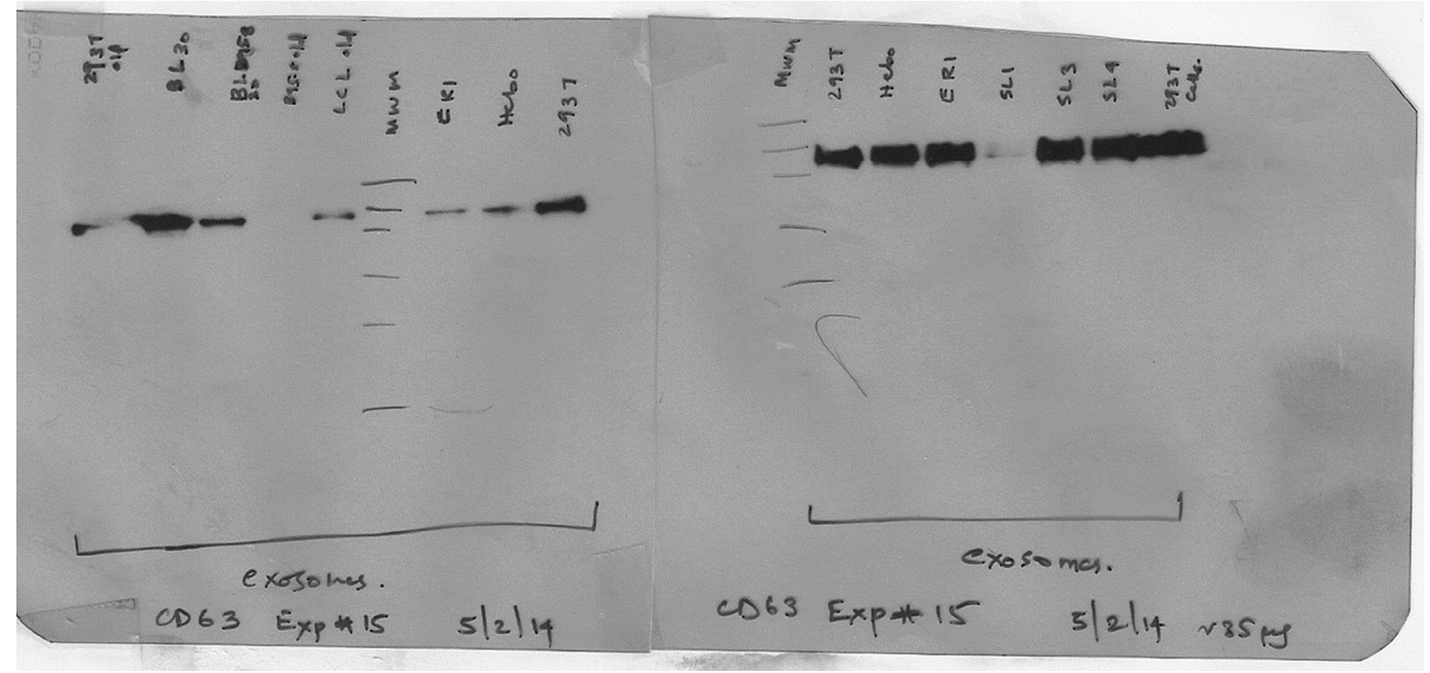
**

Supplement: Figure S2 — Western blots for CD63. Some representative western blots for CD63 on (A) cellular and (B) exosomal extracts. For the detection of CD63 in cellular extracts, 100 µg of proteins was used and for exosomal extracts 25–35 µg of protein was used. Anti-CD63 antibody (Abcam, UK) was used at a dilution 1∶1000 in non-reducing conditions. The hand written annotations refer to the various cell lines used (see main manuscript for further details). Cell lines SL1, SL3 and SL4 (Fig.B right side of the blot) are not part of this study and should not be considered here. Also note that in Fig.B (left side of the blot), exosomal extracts from both freshly prepared and older preparations were used. The relatively weak bands seen for ‘old’ samples appear to be due to protein degradation. Indeed, when freshly prepared exosomal extracts were used, stronger signals were observed e.g. compared 293T old cell extracts (left side of blot) and freshly prepared 293T cell extracts (right side of the blot) (also see Figure 3B in manuscript). (DOCX) [file pone.0099163.s002.docx]
